# Headache disorders in patients with Ehlers-Danlos syndromes and hypermobility spectrum disorders

**DOI:** 10.3389/fneur.2024.1460352

**Published:** 2024-11-08

**Authors:** Dwij Mehta, Lucy Simmonds, Alan J Hakim, Manjit Matharu

**Affiliations:** ^1^Headache and Facial Pain Group, University College London (UCL), Queen Square Institute of Neurology and The National Hospital for Neurology and Neurosurgery, London, United Kingdom; ^2^The Harley Street Clinic, HCA Healthcare, London, United Kingdom

**Keywords:** Ehlers-Danlos syndrome, hypermobility spectrum disorder, headache, migraine, CSF leak, postural tachycardia syndrome, POTS, craniocervical instability

## Abstract

Extra-articular symptoms, including headaches, are frequently encountered in patients with Ehlers-Danlos syndrome (EDS) and hypermobility spectrum disorders (HSD), and may be the presenting complaint. Migraine is reported in up to three quarters of patients with symptomatic joint hypermobility, have a higher headache frequency, and an earlier age of onset compared to the general population. Orthostatic headache is an important presentation, and should raise suspicion of an underlying spinal cerebrospinal fluid leak, dysautonomia, and craniocervical pathology, which are all associated with heritable connective tissue disorders (HCTD) including EDS. Any proposed invasive procedure should be scrupulously balanced against its potential risks, taking into account the type of EDS (e.g., vascular EDS) and its systemic manifestations. This is particularly pertinent when suspecting craniocervical instability since it remains a controversial diagnosis with a limited treatment evidence-base. This article reviews the commonly encountered headache disorders in patients with joint hypermobility-related conditions with a focus on EDS and HSD, describes their diverse presentations, and an overview of the recommended management strategies. It also emphasises the need for increased awareness of comorbid conditions in EDS and HSD among clinicians treating headaches to ensure a patient-tailored approach and facilitate a multidisciplinary approach in managing often complex cases.

## Introduction

The Ehlers-Danlos syndromes (EDS) are a heterogenous group of heritable connective tissue disorders (HCTDs) characterised by excessive joint laxity and instability, musculoskeletal pathologies including joint and soft tissue injury and skeletal deformities, skin hyperextensibility and ease of injury, and other tissue fragility including visceral organ and vascular rupture. The 2017 update in nosology identifies 13 types based on major and minor phenotypic features and pathological genetic variants that affect collagen and extracellular matrix related proteins (1). Hypermobile EDS (hEDS) is the most common type of EDS, accounting for up to 90% of cases (2). Although hEDS appears to have an autosomal dominant pattern of inheritance, unlike the other types of EDS no pathological gene variants have yet been identified and clinical findings form the basis for the diagnosis (1).

Diagnosis of hEDS requires evidence of generalised joint hypermobility and two or more of: (A) systemic manifestations of a generalised HCTD; (B) positive family history of hEDS; and (C) evidence of musculoskeletal complications. Diagnosis also requires the exclusion of other mimics, including other forms of EDS and HCTDs. For a larger group of patients who have symptomatic joint hypermobility but do not satisfy the diagnostic criteria for EDS, including hEDS, the term hypermobility spectrum disorders is used (HSD) (1).

HSD is divided into four categories: (A) generalised (G-HSD); (B) peripheral (P-HSD); (C) localised (L-HSD); and (D) historic (H-HSD). G-HSD is characterised by evidence of generalised hypermobility, which is assessed on the Beighton scoring system. Joint hypermobility limited to hands and feet is labelled as P-HSD, while involvement of a single joint, or group of joints in the same region, is labelled as L-HSD. Patients who self-report historical generalised joint hypermobility using the five-point questionnaire (5PQ) without current evidence of it on Beighton scoring system are classified as H-HSD. All subtypes require presence of at least one secondary musculoskeletal manifestation which include joint subluxations and dislocations, impaired proprioception, persistent/chronic pain, early joint degeneration, and evidence of soft tissue injury (3).

The combined prevalence of EDS and HSD is estimated to be 1 in 500, with HSD being more common than EDS (4, 5).

Clinical presentation is not limited to musculoskeletal symptoms (6). Patients frequently experience extra-articular manifestations of hEDS and HSD which include neurological (e.g., headache disorders), autonomic (e.g., postural tachycardia syndrome (PoTS)), psychological (e.g., anxiety and depression), gastrointestinal (e.g., abdominal pain, and slow gut transit), genitourinary (e.g., pelvic-girdle pain, and prolapse), cardiovascular (e.g., mitral valve prolapse), respiratory (e.g., dyspnoea, and apnoea), immunological (e.g., mast cell activation syndrome), and multisystemic (e.g., fatigue) presentations (7, 8).

Headaches are not only common in patients with hEDS and HSD but are frequently the presenting complaint and the cause of significant disability. One study reported almost a third of the hypermobile cohort had a head and/or neck symptom as their primary complaint, with migraine being the commonest headache type accounting for 82.5% of the diagnoses (9). Headache disorders are ranked third by the World Health Organisation for the overall neurological disease burden (10). They are a high additional burden of disability in a population that is frequently young and affected by multiple other systemic concerns.

The timely and accurate diagnosis, along with early introduction of treatment is hindered by a gap in knowledge and limited awareness among clinicians regarding the various headache disorders associated with joint hypermobility. This article aims to provide a clinical overview of the headache disorders frequently associated with joint hypermobility and describe practical approaches to their management.

## Headache disorders in hEDS and HSD

### Migraine

Migraine is diagnosed using the International Classification of Headache Disorders 3 (ICHD-3) and is sub-divided based on whether attacks are associated with an aura and their frequency. Headache attacks occurring on ≥15 days per month for more than 3 months, with migrainous features on at least 8 days per month, is classified as chronic migraine.

Migraine attacks can be divided into four phases, based on their temporal relationship to the headache phase. First is the premonitory phase, which is reported by around 80% of the patients and can occur up to 2 days prior to the headache. It encompasses a variety of symptoms including mood disturbance and irritability, yawning, polyuria, food cravings, photosensitivity, and neck pain.

The next phase is aura, which can precede or accompany the headache phase. The underlying mechanism remains a subject of debate, but the most widely accepted hypothesis is that of a cortical spreading depolarisation. Aura affects around 30% of the patients and tends to have a gradual evolution over 5–30 min. Typically, an aura should not last over an hour. Types of auras includes visual, sensory, language, motor, brainstem and retinal (11, 12).

The headache phase, mediated via the trigeminovascular pathway, is characterised by a throbbing or pulsatile headache that is often unilateral and is aggravated by movement. It is associated with symptoms of nausea, vomiting, photophobia and phonophobia. Headaches typically last between 4 to 72 h. The migraine attack ends with the postdromal phase, where over 80% of patients report symptoms in the 24 to 48 h following resolution of headaches. This includes fatigue, difficulty concentrating, and neck stiffness (12).

A global review of headache disorders reported migraine prevalence to be 14% (13). Chronic migraine affects approximately 2% of the population (14). Prevalence of migraine in hypermobile patients is markedly higher than the general population with estimates between 40 and 75% (15–17). A retrospective study reviewing self-reported symptoms and comorbidities of 733 patients from an EDS clinic noted migraine in 42.5% of patients with HSD/hEDS, and chronic migraine in 13.8%. In patients who had both HSD/hEDS and fibromyalgia, migraine was reported in 63.8% and chronic migraine in 34.1% (18). Acknowledging the limitations of this study, it highlights the high disease burden from headache disorders in hypermobile patients. Hypermobile patients have also been reported to experience a greater frequency of attacks, have a greater associated disability, and a younger age at onset of migraine compared to the general population (19).

Management of migraine is multifaceted. Broadly, it can be divided into non-pharmacological treatment, pharmacological treatment, and neuromodulation. Non-pharmacological options include lifestyle modification, trigger avoidance (e.g., sleep deprivation, dehydration, missed meals) and neuropsychology. Pharmacological treatment options are divided into abortive and preventative treatment. Abortive treatment consists of combination therapy in the form of a triptan, anti-emetic (prokinetic agent) and a non-steroidal anti-inflammatory drug (NSAID)/paracetamol. With the recent advent of drugs targeting calcitonin gene-related peptide (CGRP), CGRP receptor antagonists (e.g., Rimegepant) also have a role in abortive management, particularly for those patients for whom triptans are contraindicated.

Hypermobile patients are at risk of developing medication overuse headache (MOH) due to a higher prevalence of headache disorders and presence of comorbid non-headache pain conditions (e.g., widespread musculoskeletal pain, abdominal, and pelvic pain). MOH is a headache occurring on 15 or more days per month in a patient with pre-existing primary headache disorder that develops as a result of regular analgesic overuse. Use of opiates/triptans on 10 or more days per month and/or paracetamol/NSAIDs on 15 or more days per month predisposes to MOH. MOH can contribute to the transformation of episodic migraine into chronic. In fact, approximately 50% of patients diagnosed with chronic migraine would revert to episodic migraine on analgesic withdrawal (20). CGRP receptor antagonists may be a useful option in patients who do use abortive agents frequently for their migraine since they have not been associated with the development of MOH (21).

Treatment of MOH involves complete cessation of the causative analgesic(s) and initiation of preventative treatment for the underlying primary headache disorder, if not already done so. In the absence of robust evidence to guide the speed of treatment withdrawal, the authors recommend a gradual approach in patients where the offending drug is an opiate as the withdrawal symptoms can be significant (22). It is important to warn the patient that the headache may initially worsen before improving regardless of whether the cessation is gradual or abrupt. Withdrawal headache can last between 2 and 10 days from time of complete withdrawal. A greater occipital nerve (GON) block to bridge this period may be helpful. Improvement of baseline headache can take up to 3 months.

Preventative treatment is considered in patients with >3 headache days/month, or highly disabling headaches. The aim of preventative treatment is to reduce the frequency and severity of headaches. Complete resolution of headaches is unrealistic and should be discussed with patients. The authors recommend continuing a target dose for at least 3 months, unless side-effects intervene. If efficacious (>30% improvement in severity and/or frequency), a patient can be weaned off the drug after 12 months.

It is imperative comorbidities are considered when prescribing a preventative agent. This is particularly true in hypermobile patients due to associations with dysautonomia (e.g., PoTS and Raynaud’s phenomenon), mood disorders, gastrointestinal disorders (e.g., slow transit constipation), and cognitive perturbance, which can contraindicate the use of several commonly used agents (Table 1).

**Table 1:** Migraine preventative agents and their side-effect profiles.

	Drug	Max dose	Side-effects/ cautions
Anti-epileptic	Topiramate	100 mg BD	Weight loss, cognitive blunting, anxiety/ depression, renal calculi, angle-closure glaucoma.
	Sodium valproate	1,000 mg BD	Weight gain, teratogenicity, tremor, alopecia, thrombocytopaenia, hepatotoxicity, hyperammonaemia.
Antidepressants	TCA (amitriptyline/ nortriptyline)	75 mg/ 75 mg	Constipation, urinary retention, orthostatic hypotension, drowsiness, dry mouth, dry eyes, prolong QTc interval.
	SNRI (venlafaxine)	150 mg BD	Constipation/ diarrhoea. Use with caution in elderly, patients with cardiac disease, epilepsy, uncontrolled hypertension and angle-closure glaucoma.
Anti-hypertensives	ACE-inhibitor (Lisinopril)	20 mg OD	Orthostatic hypotension, dry cough, angioedema, hyperkalaemia, renal impairment.
	ARB (Candesartan)	8 mg BD	Orthostatic hypotension, vertigo, cough, hyperkalaemia, renal impairment.
Beta-blockers	Propranolol	240 mg/day	Fatigue, impotence, mood disorder, bradycardia. Avoid if history of asthma/ heart block/ bradycardia.
	Atenolol	200 mg/day	
	Metoprolol	200 mg/day	
Others	Pizotifen	3 mg OD	Weight gain, dry mouth, constipation. Caution in patients with epilepsy/ angle closure glaucoma/ urinary retention.
	Calcium channel blocker (Flunarizine)	10 mg OD	Weight gain, depression and extrapyramidal side-effects.
Injectables	Botulinum Toxin Type A	155–195 units, three monthly (intramuscular)	Transient neck pain, muscle weakness, ptosis.
CGRP monoclonal antibodies	Erenumab	70-140 mg monthly	Constipation, vertigo, hypersensitivity reactions, nasopharyngitis.
	Fremanezumab	225 mg monthly/ 675 mg three monthly	
	Galcanezumab	120-240 mg monthly	
	Eptinezumab	100-300 mg three monthly (intravenous)	
Gepants	Atogepant	60 mg OD	Fatigue, constipation, nausea, reduced appetite, hypersensitivity reaction.
	Rimegepant	75 mg on alternate days	
Supplements	Coenzyme Q10	100 mg TDS	Mild gastrointestinal symptoms.
	Magnesium	600 mg daily	With toxicity, can cause diarrhoea and arrythmia.
	Riboflavin	400 mg/day	Nil.

OD, once daily; BD, bis die (twice daily); TDS, ter die sumendum (three times daily); TCA, tricyclic antidepressant; SNRI, serotonin and norepinephrine reuptake inhibitors; ACE, angiotensin-converting enzyme; ARB, angiotensin 2 receptor blocker; CGRP, calcitonin gene receptor peptide.

Lastly, neurostimulation forms the final part of migraine management algorithm. Within non-invasive neurostimulation options, external trigeminal neurostimulation using the Cefaly device and single pulse transcranial magnetic stimulation (sTMS) can be utilised for abortive treatment, with recent evidence also supporting their uses for migraine prevention. Occipital nerve stimulation (ONS) has a role as preventative treatment in chronic migraine. ONS involves implantation of suboccipital electrodes that are connected to an implanted pulse generator in the chest or abdomen. Potential complications with ONS include infection, skin erosion, lead migration, lead fracture, persistent pain around implant site, haemorrhage, and nerve damage. Careful consideration is needed when pursuing ONS in a subset of hypermobility patients with poor wound healing and tissue fragility due to risk of significant complications.

### Orthostatic headaches

Headaches are described as orthostatic when there is a postural component to them. The headaches are exacerbated or triggered by assumption of an upright position, and either improve or resolve with recumbency. Although there is currently no consensus or evidence-base for how rapid the onset and/or offset needs to be for it to constitute an orthostatic headache, headache of this quality in a patient raises the possibility of cerebrospinal fluid (CSF) hypovolaemia (spontaneous or iatrogenic), PoTS, craniocervical instability (CCI), migraine, or paradoxical presentation of idiopathic intracranial hypertension (IIH) (23). Except for iatrogenic CSF leak, all of these differential diagnoses (Table 2) are associated with hEDS/HSD and are therefore of great relevance when reviewing a hypermobile patient with headaches (24).

**Table 2:** Differentials for orthostatic headaches in patients with EDS.

Condition	Clinical note	Diagnostic note
Spontaneous intracranial hypotension	Thunderclap onset Consistent onset and offset times with change in posture End-of-day headache worsening Headaches induced or exacerbated by Valsalva manoeuvres Behavioural variant FTD-like presentation	Bilateral subdural collections Diffuse smooth pachymeningeal enhancement Brain sagging Distended dural venous sinuses Spinal epidural CSF collection Infratentorial superficial siderosis
Postural tachycardia syndrome	Symptoms of orthostatic intolerance (e.g., lightheadedness, syncope, palpitations, dyspnoea, chest pain, tremulousness) Preceding infectious trigger, or prolonged bedrest Symptoms worst on awakening and exacerbation by heat, fever, and dehydration	Abnormal active stand test Positive tilt table test, and negative MRI for evidence of SIH
Chronic migraine	Motion sensitivity rather than a postural component Gradual increase in frequency of previous episodic migraine	Clinical diagnosis, with exclusion of other differentials.
Craniocervical instability	Symptoms attributable to brainstem dysfunction (e.g., diplopia, dysphagia, sleep apnoea, sensorimotor disturbance affecting upper and/or lower limbs).	Abnormal dynamic and/or upright CT/MRI in-keeping with craniocervical instability.

FTD, frontotemporal dementia.

#### Postural tachycardia syndrome

The association between joint hypermobility and dysautonomia is increasingly recognised (25, 26). Presentations of dysautonomia include PoTS, orthostatic intolerance, neuro-cardiogenic syncope, orthostatic hypotension, and Raynaud’s phenomenon. Prevalence of dysautonomia in EDS has been reported to be as high as 78% in the literature, highlighting the enormity of the problem among hypermobile cohort (27). One study found 71% of the EDS cohort that suffered from headaches also had a diagnosis of autonomic dysfunction, suggesting a possible causal association (28).

Of the various presentations of dysautonomia, PoTS in particular is associated with orthostatic headaches (29). It represents a highly disabling condition that is defined by a combination of exaggerated increase in heart rate on standing and associated symptoms of orthostatic intolerance. Diagnosis requires a sustained rise in heart rate of >30 beats per minute (bpm) in adults, and over 40 bpm in adolescents and children (<20 years of age), within 10 min of standing or head-up tilt (HUT). Symptoms of orthostatic intolerance include headache, light headedness, presyncope, palpitations, chest pain, dyspnoea, and tremulousness. There may be a preceding history of viral illness (up to 20–50%), vaccination, prolong period of bedrest, or a history of joint hypermobility (30, 31). Symptoms are frequently worse on first waking up and are exacerbated by hot temperature, fever, dehydration, and standing up for long duration.

PoTS has long been listed as a differential for patients presenting with orthostatic headache and has also been well described in the scientific literature. A prospective study of 24 consecutive patients with PoTS evaluated the presence of headaches during HUT. Fourteen patients had orthostatic headaches during daily activity, while 15 developed orthostatic headaches during HUT (29). Heyer and colleagues also proposed orthostatic headache as a predictive symptom for PoTS. In this study, authors reviewed adolescents referred to the unit for tilt-table testing and found that 33 out of 37 patients with PoTS had an orthostatic headache, compared to seven out of 33 patients without PoTS. This translated to pre-test sensitivity of 89.2% and specificity of 78.8% (32). In a systematic review by Ray et al., the prevalence of orthostatic headache in PoTS was reported in only four eligible studies and ranged between 2.2–58.3% (33).

The exact mechanism behind increased prevalence of PoTS in hypermobile patients remains elusive. One of the more accepted hypotheses relates to excessive distensibility of the veins, due to the underlying HCTD, leading to venous pooling and reduced venous return. This in turn results in a lower cardiac output stimulating reflex tachycardia and orthostatic intolerance. Due to the increased prevalence of small fibre neuropathy in EDS, this has also been speculated to be a potential cause of dysautonomia. Other possible causes include a hyperadrenergic state, excessive histamine-induced vasodilation possibly in context of comorbid mast cell activation syndrome, as well as use of vasoactive drugs (e.g., tricyclic antidepressants and opiates) again due to comorbid conditions in hypermobile patients. The pathophysiological basis of headache in PoTS is even less clear and there is need for further research (34, 35).

In hypermobile patients presenting with an orthostatic headache, there should be a low threshold in screening for PoTS. An active stand test is a simple to do and effective. It involves checking heart rate and blood pressure after lying flat for at least 15 min, before serial measurements on becoming upright after 0, 2, 5, and 10 min. The patient is asked to stand quietly and remain still. Due to recognised limitations, including the influence of factors such as fluid and fasting status, and intake of caffeine or nicotine, the authors’ practice is to acquire multiple readings over multiple days and different times of the day. If the readings approach the arbitrary threshold for a diagnosis of PoTS, and self-management treatments do not sufficiently help, patients are referred to a specialist autonomic or cardiology service for tilt-table testing to facilitate a formal diagnosis and instigate appropriate management.

The lack of research in the field means there is no evidence-base to guide use of specific headache treatments in people with orthostatic headache. Management is directed at optimising PoTS through non-pharmacological and pharmacological interventions. Non-pharmacological interventions include expansion of intravascular volume (intake of 2–3 litres of water and 10–12 grams of salt per day), lifestyle modification (limit amount of time spent lying down, and improve sleep hygiene), functional movement and other exercise programmes, and use of compression clothing (33).

Pharmacological treatment is aimed at management of symptoms rather than disease-modification. Medications are divided by mechanism of action into volume expanders, negative chronotropes, vasopressors, and sympatholytic agents. Medication choice is tailored to the needs of each patient. For example, propranolol is considered if tachycardia and palpitations are the prominent symptoms, whereas midodrine might be considered in those with a less severe tachycardia and where light-headedness from hypotension rather than palpitations is the predominant concern (36). From the authors’ experience, orthostatic headache often, but not invariably, improves with optimisation of PoTS, however, as this is a chronic condition it is also not uncommon for the headaches to flare-up during exacerbations of PoTS.

#### Spontaneous intracranial hypotension

Spontaneous intracranial hypotension (SIH) is caused by the spinal escape of CSF resulting is CSF hypovolaemia. Three causes are currently recognised: ventral dural tear secondary to herniated calcified disc or an osteophyte, spinal nerve root sleeve leak, and CSF venous fistula (CVF).

The incidence of SIH is estimated to be 5 per 100,000 with a female predilection (2:1) (37). The presence of a HCTD, such as hEDS is considered a risk factor for developing SIH. In one prospective study 18% of the cohort (*n* = 50) had a HCTD, while another 16% were found to have ‘benign joint hypermobility syndrome’ (BJHS) or isolated features of the HCTDs (38).

Patients typically present with an orthostatic headache, although other headache presentations are well recognised, including second-half-of-the-day and Valsalva-induced headaches. The orthostatic component can be lost with increasing chronicity of the condition and as such the headache history at initial presentation must be carefully revisited and phenotyped to avoid misdiagnosis. Besides headache, migrainous, audiovestibular, cognitive, and musculoskeletal symptoms are very common. Untreated, potential complications include superficial siderosis, bibrachial amyotrophy, cerebral venous sinus thrombosis, and frontotemporal brain sagging syndrome.

The initial work-up consists of contrast-enhanced brain MRI and whole spine MRI for evidence of CSF hypovolaemia and a spinal longitudinal epidural collection (SLEC), respectively. It is recognised that brain MRI can be normal in up to 20% of cases and thus normal MRI does not exclude SIH. As per the consensus guidelines by Cheema and colleagues, we advise against the use of a lumbar puncture (LP) when investigating for SIH since only a third of patients have a low opening pressure of <6 cmH_2_O, and it risks further exacerbation of symptoms through a post-dural puncture CSF leak (39).

When considering targeted management, localisation of the site of CSF escape is necessary. While standard MRIs have no localising value, they do help in predicting the underlying aetiology. SLEC is usually seen in ventral dural tears and proximal nerve root sleeve leaks and is absent in CVF and distal nerve root sleeve leaks. In turn, this determines the positioning of the patient when proceeding with localising investigations in the form of CT or digital subtraction myelography. Prone position is used for patients with a SLEC, while a lateral decubitus position is utilised in those without. Due to the suboptimal sensitivity of myelographic studies, it is not uncommon to have to repeat them on multiple occasions.

There is a subset of patients, often hypermobile, who have orthostatic headaches and symptoms highly suggestive of SIH, where PoTS has been excluded, but both MRIs and myelograms are consistently negative for evidence of CSF escape. The underlying pathophysiological mechanisms driving the symptoms remain unknown but may relate to increased compliance of the spinal compartment, or reduced CSF outflow resistance (40). Alternatively, a proportion of patients may have an occult CSF leak or fistula. This is supported by the findings from Schievink and colleagues who reported 10% of patients with orthostatic headaches and normal brain MRI and conventional spinal imaging had an underlying CVF on digital subtraction myelogram (41).

Treatment options are divided into conservative and invasive. Conservative management includes bed rest, adequate hydration (2–2.5 litres per day), use of caffeine (oral and/or intravenous), analgesics and abdominal binders, and avoidance of Valsalva manoeuvres. Invasive treatment can be subclassified into non-targeted epidural blood patch (EBP) and targeted treatment. The authors recommend persevering with conservative management for no longer than 2 weeks after the onset of symptoms as it has a relatively low success rate; estimated to be 28% in one meta-analysis (42). It is also recognised that early treatment is associated with improved outcomes (43). If symptoms persist non-targeted EBPs may be recommended. Meta-analysis suggests first epidural blood patch leads to resolution of symptoms in 64% of patients, but this number drops sequentially with repeat procedures. The authors therefore limit EBPs to two and if there is ongoing evidence of CSF hypovolaemia proceed to myelographic studies for localisation (39). If and when the CSF leak is localised, targeted management is pursued, and the type of treatment is dependent on the type of CSF leak. This includes targeted epidural blood patch, fibrin patch, transvenous embolisation, and surgical management.

#### Craniocervical instability

The craniocervical junction (CCJ) comprises of the occiput, atlas, axis, and its associated group of muscles and ligaments that under normal circumstances allow for a high range of movement while ensuring structural integrity to avoid risk of damage to adjacent nerve roots or the spinal cord (44–46).

CCI is most commonly recognised in context of trauma but is also seen as part of congenital osseous malformation and HCTDs. EDS has recently gained significant attention as a risk factor for the development of CCI. The proposed mechanism behind CCI in EDS has been attributed to excessive ligamentous laxity at the CCJ resulting in atlantoaxial subluxation and cranial settling. This is thought to cause compression and injury to neurological structures near the brainstem, leading to a wide array of symptoms that constitute the cervico-medullary syndrome (CMS) (47–49).

The prevalence of orthostatic headache in CCI is unknown despite CCI being widely considered as a differential diagnosis in orthostatic headaches. The small numbers of published reports also do not phenotype the headaches in detail, preventing meaningful comparison with other causes of orthostatic headaches to help deduce any disease-specific headache characteristics.

When CCI is suspected, a patient should be assessed by a specialist neurosurgical team with experience in managing this condition. Diagnosis is made based on radiological parameters thought to reflect CCI in context of symptoms consistent with CMS. Given CCI symptoms in EDS are often positional and exacerbated by upright posture, dynamic and/or upright (weight-bearing) imaging is performed in the form of CT and MRI (47, 50). Management is frequently guided by symptom severity and associated disability (51). First-line is conservative management (e.g., cervical orthosis and physiotherapy). A lack of response and/or progression of symptoms would be one of the indicators for considering surgical management (48).

CCI as an entity remains a highly controversial topic. There is no way to differentiate between hypermobility and instability (47). The specific radiological measurements used to make the diagnosis are inconsistently applied across the different centres that manage this condition, while no normative data currently exists for these measurements. There is a need for a study in healthy subjects using structural MRI to establish normative data for the commonly used morphometrics in diagnosing CCI. Additionally, further studies utilising upright dynamic MRI are required to ascertain whether hypermobile patients experiencing headaches and/or symptoms potentially related to the brainstem (CMS) exhibit evidence of a hypermobile craniocervical junction, or brainstem compression, compared to hypermobile patients lacking such symptoms.

There is also a lack of signal change seen within the spinal cord on MRI of the patients labelled as CCI to support the current proposed pathophysiology. Furthermore, a significant overlap exists between EDS, PoTS, and migraine. All the symptoms attributed to CCI can potentially be explained by these diagnoses. Thus, CCI is an area that urgently requires further research to ensure there is a strong evidence-base for the diagnosis and management in hypermobile patients.

### Cervicogenic headaches

Cervicogenic headache is a secondary headache disorder occurring as the result of a cervical pathology. Recognised causes include, but are not limited to, facet arthropathy, fracture, local infectious and malignant processes (52). Cervical hypermobility and its secondary effects, with or without atlantoaxial instability, have been proposed as the possible mechanism behind cervicogenic headaches in patients with EDS or HSD. Scoliosis is associated with joint hypermobility, and it may also play a role in subjecting the cervical spine to supranormal stress, predisposing to cervical spondylosis and disc herniation, with resultant cervicogenic headache (53).

Headache is frequently centred over the occipital region and is chronic. Neck pain is invariably present, although not necessary for the diagnosis, and there may be associated pain radiating to the shoulder and ipsilateral arm (53, 54). ICHD-3 diagnostic criteria requires a temporal relationship between the headache and the onset of a cervical disorder that is recognised to cause headache, and to improve substantially on resolution of the pathology. Accompanying reduced range of movement and provocation of pain with neck movement are supportive features. Finally, absolute response to diagnostic block is part of the diagnostic criteria (52).

Within the neurology community, diagnosis of cervicogenic headache is controversial. Frequently, the presence of neck pain, in context of cervical tenderness to palpation and exacerbation by neck movement, radiological evidence of spondylosis, or good response to GON/diagnostic nerve blocks are deemed sufficient for the diagnosis of cervicogenic headache. However, it is not without its pitfalls and the diagnosis requires careful consideration as to avoid misdiagnosing patients where a primary headache disorder may be the more likely cause. For example, neck pain is a very common finding in primary headache disorders with a prevalence in migraine of up to 70% (55). Cervical tenderness to palpation has been shown to have poor diagnostic value and myofascial tenderness is frequently seen in patients with migraine (56, 57). Furthermore, a study found no difference in incidence of spondylosis on imaging between patients diagnosed with cervicogenic headache and healthy controls (58). Lastly, response to GON blocks is not specific to cervicogenic headaches and has no diagnostic or localising value. Their role in management of primary headache disorders is well established, including migraine and cluster headaches.

Therefore, while there are anatomical and pathophysiological bases for cervicogenic headaches, associated clinical and radiological features, alongside response to GON blocks, are not specific to the disorder and can frequently be found in, and explained by, other primary headache disorders. This is particularly important in the hypermobile cohort where primary headache disorders such as migraine are highly prevalent.

### Temporomandibular joint disorders

Temporomandibular joint disorders (TMD) consist of a group of conditions affecting either the temporomandibular joint (TMJ) or the masticatory muscles. Its incidence peaks between the ages of 20–40 years and has a female predominance (59).

Risk factors for development depends on the type of TMD: intraarticular (TMJ) versus myofascial (masticatory muscles). Risk factors for an intraarticular TMD include articular disc displacement, trauma, osteoarthritis, and inflammatory causes. Joint hypermobility is associated with intraarticular TMD, possibly because of ligamental laxity causing TMJ subluxation, or even complete joint dislocation. Myofascial TMD risk factors include bruxism, mood disorders, autoimmune conditions, and chronic pain disorders. HCTDs, in addition to contributing to intraarticular TMDs, are also associated with a higher prevalence of mood disorders and chronic pain disorders, and may be implicated in the development of both intraarticular and myofascial TMDs (60).

Symptoms of TMDs include pain, TMJ clicking (also crepitations and popping with opening/closing of mouth), limitation of jaw opening, and audiovestibular symptoms (vertigo, tinnitus, and aural fullness). Pain is frequently centred over the temporal region, occipital region, periauricular region, neck and over the TMJ. Pain is typically triggered by function, such as swallowing, talking, and chewing. Palpation can also reveal tender regions (59, 61). TMDs are also associated with an increased prevalence of migraine, chronic daily headaches, and possibly episodic tension type headache (62).

Patients should be assessed by specialists with experience in TMD management such as oral medicine clinicians, oral and maxillofacial surgeons, and ear, nose and throat surgeons. MRI is the gold standard imaging modality for assessment of disc displacement and soft tissue (e.g., synovium and lateral pterygoid muscle) changes. Other imaging modalities include panoramic radiography, plain radiographs, CT and high-resolution ultrasonography (63). Management is multifaceted and includes conservative treatment (e.g., reassurance, patient education, soft diet), physiotherapy (e.g., jaw stretching exercises, posture training), psychology (e.g., cognitive behavioural therapy), pharmacotherapy (e.g., NSAIDs, benzodiazepines, neuropathic agents) and invasive treatment (e.g., intraarticular corticosteroid injection, Botox injections). Conservative management can improve symptoms in 50–90% of patients, while up to 40% of patients gain remission without any intervention (59, 60, 64).

### Tension-type headache

Tension-type headache (TTH) is a primary headache disorder characterised by episodes of largely featureless bilateral headaches that are of a mild-to-moderate intensity with a tightening or pressing quality. Under the ICHD-3 diagnostic criteria, it is subclassified based on its frequency (infrequent episodic, frequent episodic, and chronic) and whether it is associated with pericranial tenderness.

TTH is reported to have a global 1-year prevalence of 26.8%, which is higher than that of its main differential, migraine. It is differentiated from migraine through its lack of association with nausea, photophobia and phonophobia. Furthermore, unlike in migraine, autonomic symptoms and aggravation of headache by physical activities are uncommon in TTH.

In clinical practice, distinguishing between the two conditions can be challenging, especially in patients who present with both headache types. Currently, there are no disease-specific radiological investigations or biomarkers, and diagnosis of TTH is based solely on clinical history. Congruent with our own experience, the Spectrum study has reported that a large proportion of headaches labelled as TTH are in fact migraine headaches when assessed by a neurologist, highlighting the importance of carefully reviewing the headache phenotype.

A Danish epidemiologic study identified poor self-rated health, inability to relax after work, few hours of sleep per night, female sex, and young age as risk factors for TTH. Unlike migraine, studies have not shown an increased prevalence of TTH in the hypermobile population. An Iranian observational-analytical study found no significant difference in the frequency of TTH between patients with BJHS and healthy controls. In a retrospective study by Malhotra and colleagues, only 2/140 patients with joint hypermobility had TTH. Similarly, Bendik et al. (17) observed a comparable prevalence of TTH in hypermobile patients and healthy controls but noted a higher TTH attack frequency in the hypermobile cohort. However, the latter study was relatively small, with only 28 hypermobile patients, preventing any definitive conclusions to be drawn.

Due to its milder severity when compared to migraine, patients are less likely to seek medical attention for its management. When management is required, simple analgesics can be utilised for abortive treatment. It is again imperative to consider risk of MOH and this should be discussed with patients. In patients with chronic TTH, oral preventative treatment can also be considered (e.g., amitriptyline or mirtazapine).

### Chiari malformation 1

Chiari malformation 1 (CM-1) is the most common type of Chiari malformation with an estimated prevalence of up to 3.6% in MRI-based studies (65). CM-1 is characterised by an abnormally shaped cerebellar tonsils that are displaced below the level of foramen magnum. The general consensus among specialists is that a cerebellar tonsillar displacement of ≥5 millimetres below the foramen magnum is required for a radiological diagnosis (66). Mesodermal or neuroectodermal anomalies form the aetiological basis for the development of CM-1 (67). Milhorat and colleagues reported an overlap between CM-1 and HCTDs with 12.7% of their 2,813 patients with CM-1 meeting the diagnostic criteria for HCDTs, hEDS accounting for over 41% of this overlapping cohort (68).

There is a reported higher female-to-male ratio among CM-1 patients with a background of HCTD, compared to those without. Symptom onset may also be significantly earlier in patients with HCTDs who may first develop symptoms in adolescence (69).

CM-1 can manifest clinically in a variety of ways including headaches, syrinx formation, brainstem syndrome, cerebellar dysfunction, and occasionally hydrocephalus. CM-1 can also be an incidental finding on MRI with no attributable symptoms.

Headache is the most common presentation in CM-1. It is present in up to 81% of patients and is often triggered by Valsalva manoeuvres such as coughing (70). Pain typically involves the occipital region and the neck, with a duration of less than 5 min. The underlying mechanism for Valsalva-induced headache is thought to relate to the perturbed CSF flow at the level of the foramen magnum secondary to tonsillar herniation, but further research is required to ascertain the exact mechanism (71). Besides Valsalva-induced headaches, migraine is reported to have a higher prevalence in CM-1 patients with HCTD than those without HCTD (69). Features of intracranial hypertension may also be present and could indicate development of hydrocephalus.

Syrinx formation is a common complication, with frequency estimated between 35 and 75% in the paediatric CM-1 population (72). It is most commonly located in the cervical spine, and can occasionally extend cranially into the medulla oblongata, resulting in both a spinal cord and a brainstem syndrome.

CM-1 evaluation consists of brain and whole spine MRI. Whole spine MRI is important in assessing for development of syringomyelia (73). Phase contrast MRI is often also utilised to assess for CSF flow across the foramen magnum, which can influence management. In patients who are asymptomatic, conservative management with MRI surveillance can be considered, whereas neurosurgical evaluation for decompressive surgery is considered in those that are symptomatic and/or have evidence of CSF flow obstruction (74, 75). It is imperative that associated systemic comorbidities are given thorough consideration when pursuing surgical management in patients HCTDs to help achieve optimal outcomes. In a patient with EDS knowledge of the specific EDS type is also necessary since elective surgery in patients with vascular EDS, for example, may have potentially life-threatening complications.

A rare, but important, differential diagnosis for CM-1 in the hypermobile cohort is SIH. SIH can present with Valsalva-induced headaches and brain MRI can show pseudo-Chiari malformation as part of brain sagging. Moreover, pseudo-Chiari malformation can also result in syringomyelia, further complicating the clinical picture. The authors recommend a detailed history, focusing particularly on whether there was an orthostatic headache at the onset of the headache. When SIH is suspected, specialist review of brain MRI for evaluation of other radiological features associated with SIH, with consideration of contrast-enhanced brain MRI and whole spine MRI, is critical before proceeding with surgical management for CM-1 (73).

### Vascular headache

Some HCTD, including some types of EDS are associated with an increased risk of neurovascular events such as aneurysmal subarachnoid haemorrhage, spontaneous arterial dissection, and carotid cavernous fistula (CCF). A patient presenting with a thunderclap headache, features of meningism, and/or impaired consciousness should raise the suspicion for subarachnoid haemorrhage. Similarly, acute onset of head or facial pain (usually ipsilateral to the dissection), associated neck pain, and focal neurology (e.g., Horner’s syndrome or relating to ischaemic stroke) are concerning features for a cervical artery dissection. Unlike the general population, patients with EDS can develop dissections spontaneously, in absence of a precipitant (1).

Vascular EDS and Marfan syndrome have been linked to the development of CCF (76). There is an aberrant connection between the carotid (internal or external) artery and the cavernous sinus. Presentation is variable depending on the type of fistula (e.g., direct versus indirect, and high versus low flow), but frequently includes headaches and ophthalmic signs. Headaches are often peri-or retro-orbital in location. Although ipsilateral pain is most common, patients can experience bilateral symptoms. Ophthalmic features include pulsatile exophthalmos, conjunctival injection, chemosis, diplopia, elevated intraocular pressure, as well as papilloedema. Early consideration of an underlying vasculopathy related to an HCTD is required as management can be high-risk due to associated vascular fragility.

### New daily persistent headache

New daily persistent headache (NDPH) is a rare headache disorder with a distinct onset that is clearly remembered by the patient and becomes continuous within 24 h of onset. The headache needs to persist for over 3 months to fulfil the diagnostic criteria for NDPH. One study estimated 1 year prevalence of NDPH to be 0.03% (77). Studies have linked underlying HCTDs to an increased prevalence of NDPH, including Rozen and colleagues who reported cervical and widespread hypermobility in 11 and 10 patients with NDPH, respectively (78). A more recent study by Cheema and colleagues comparing NDPH and transformed chronic daily headaches (T-CDH), noted a background of joint hypermobility syndrome in 11.2 and 18.5% of their NDPH (*n* = 366) and T-CDH (*n* = 696) groups, respectively (79). The vast majority of the T-CDH was composed of patients with chronic migraine (99%).

Around half of patients diagnosed with NDPH recall a triggering event such as a flu-like illness, procedures requiring intubation and period of neck extension, or a stressful event (80). Phenotypically, NDPH can bear characteristics of chronic migraine or chronic tension-type headache. Previous history of migraine or tension-type headache (TTH) does not preclude one from making a diagnosis of NDPH if the criteria for NDPH are also met and there is no history of worsening headache frequency in association with migraine or TTH. The management is guided by the dominant clinical phenotype, although NDPH is frequently refractory to the standard treatments utilised in migraine (79).

Occasionally, spinal CSF leak can present as a NDPH. Presence of an orthostatic quality should be sought when taking a clinical history for a patient with NDPH; when present, there should be a low threshold to investigate for SIH. In patients with a new unilateral daily and persistent headache with prominent autonomic features, a diagnosis of hemicrania continua (a unilateral continuous headache for more than 3 months with episodic headache exacerbations) should be considered.

The pathophysiological basis for NDPH remain poorly understood. Given the phenotypic similarities of NDPH to migraine and TTH, it has been postulated that NDPH represents de-novo, and a persistent, variant of the two conditions. Others have proposed a possible inflammatory aetiology due to the associations with infections including Epstein–Barr virus and herpes simplex virus, as well as elevated levels of tumour necrosis factor alpha in CSF of patients with NDPH (81). Lastly, as described above, cervical hypermobility has also been implicated as a potential cause of NDPH.

## Conclusion

**Figure 1:**
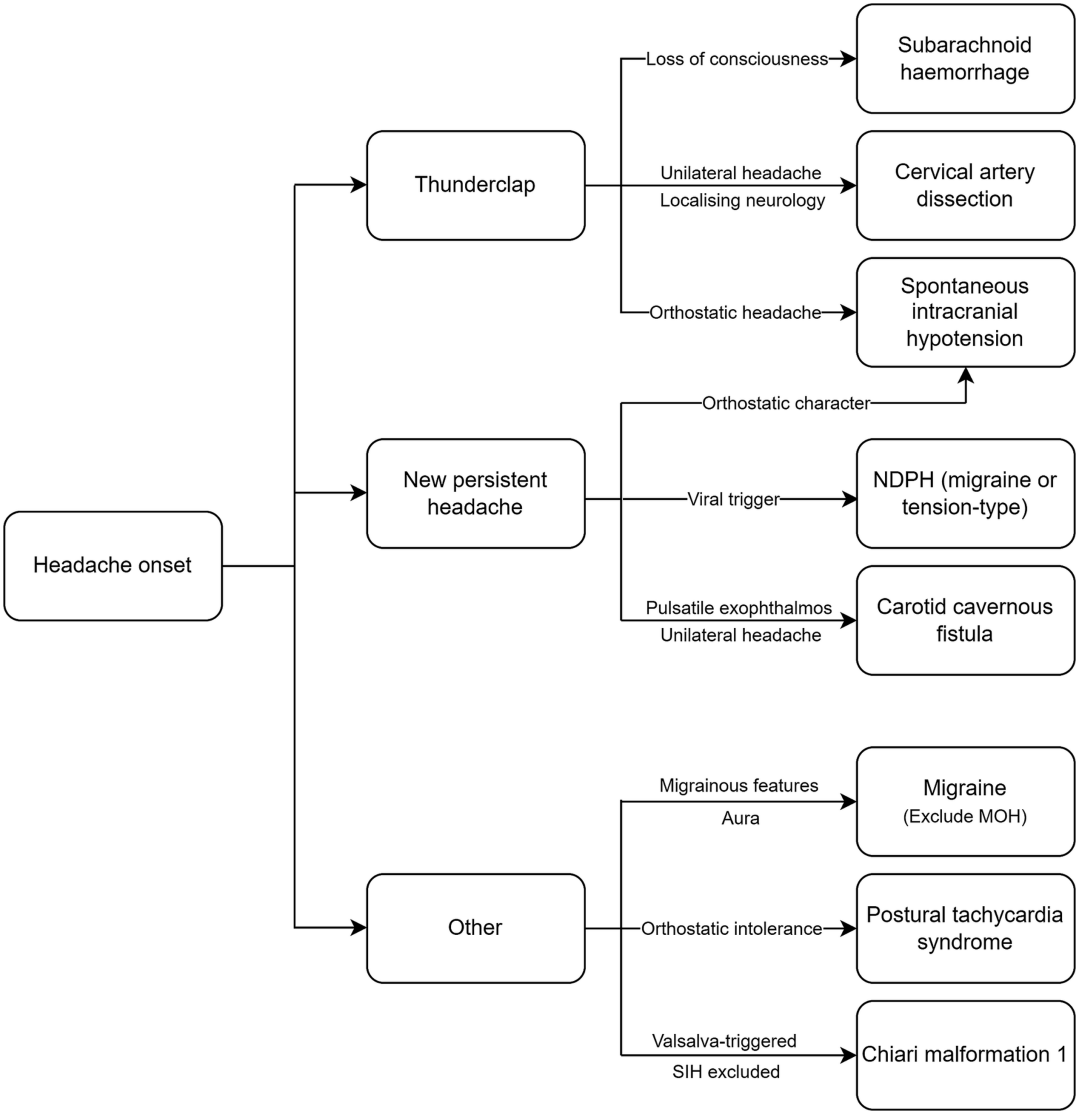
Diagnostic algorithm for headache disorders in EDS and HSD. NDPH, new daily persistent headache; MOH, medication overuse headache; SIH, spontaneous intracranial hypotension.

Headaches are among the most common extra-articular manifestations in patients with joint hypermobility-related disorders. Both primary and secondary headache disorders are associated with HSD and EDS and require careful consideration (Figure 1) to ensure an early diagnosis and the timely institution of treatment, particularly in the case of secondary headaches. When selecting a treatment option, clinicians must consider the type of EDS and systemic manifestations, as this will influence the choice of intervention. The pathophysiological basis for the increased prevalence of certain headache disorders in EDS and HSD remain poorly understood and there is a need for further research. Proposed factors include cervical hypermobility leading to instability, autonomic dysfunction causing orthostatic intolerance and altered pain perception, and vascular fragility. However, there is a lack of robust data that clearly delineates the causal mechanisms. Understanding the pathophysiology may lead to the development of targeted treatments tailored to patients with EDS or HSD, and guide interdisciplinary approaches to identifying and treating these conditions.

## References

[1] Malfait F Francomano C Byers P Belmont J Berglund B Black J. The 2017 international classification of the Ehlers-Danlos syndromes. Am J Med Genet C: Semin Med Genet. (2017) 175:8–26. doi: 10.1002/ajmg.c.31552 28306229

[2] Tinkle B Castori M Berglund B Cohen H Grahame R Kazkaz H. Ehlers-Danlos Syndrome type iii and Ehlers-Danlos Syndrome hypermobility type: clinical description and natural history. Am J Med Genet C: Semin Med Genet. (2017) 175:48–69. doi: 10.1002/ajmg.c.31538, PMID: 28145611

[3] Castori M Tinkle B Levy H Grahame R Malfait F Hakim A. A framework for the classification of joint hypermobility and related conditions. Am J Med Genet C: Semin Med Genet. (2017) 175:148–57. doi: 10.1002/ajmg.c.31539, PMID: 28145606

[4] Demmler JC Atkinson MD Reinhold EJ Choy E Lyons RA Brophy ST. Diagnosed prevalence of Ehlers-Danlos Syndrome and hypermobility Spectrum disorder in Wales, Uk: a National Electronic Cohort Study and case-control comparison. BMJ Open. (2019) 9:e031365. doi: 10.1136/bmjopen-2019-031365, PMID: 31685485 PMC6858200

[5] Carroll MB. Hypermobility Spectrum disorders: a review. Rheumatol Immunol Res. (2023) 4:60–8. doi: 10.2478/rir-2023-0010, PMID: 37637226 PMC10457547

[6] Clark NL Johnson M Rangan A Kottam L Swainston K. The biopsychosocial impact of hypermobility Spectrum disorders in adults: a scoping review. Rheumatol Int. (2023) 43:985–1014. doi: 10.1007/s00296-023-05298-2, PMID: 36894757 PMC10126066

[7] Yew KS Kamps-Schmitt KA Borge R. Hypermobile Ehlers-Danlos Syndrome and hypermobility Spectrum disorders. Am Fam Physician. (2021) 103:481–92. PMID: 33856167

[8] Hakim AJ Tinkle BT Francomano CA. Ehlers-Danlos syndromes, hypermobility Spectrum disorders, and associated co-morbidities: reports from Eds Echo. Am J Med Genet C: Semin Med Genet. (2021) 187:413–5. doi: 10.1002/ajmg.c.31954, PMID: 34793630

[9] Malhotra A Pace A Ruiz Maya T Colman R Gelb BD Mehta L. Headaches in hypermobility syndromes: a pain in the neck? Am J Med Genet A. (2020) 182:2902–8. doi: 10.1002/ajmg.a.61873 32940405

[10] Organisation WH. Headache Disorders. (2016). Available from: https://www.who.int/news-room/fact-sheets/detail/headache-disorders. (Accessed April 03, 2024).

[11] Dodick DW. Diagnosing secondary and primary headache disorders. Continuum (Minneap Minn). (2021) 27:572–85. doi: 10.1212/con.0000000000000980 34048392

[12] Recober A. Pathophysiology of migraine. Continuum (Minneap Minn). (2021) 27:586–96. doi: 10.1212/con.0000000000000983 34048393

[13] Stovner LJ Hagen K Linde M Steiner TJ. The global prevalence of headache: an update, with analysis of the influences of methodological factors on prevalence estimates. J Headache Pain. (2022) 23:34. doi: 10.1186/s10194-022-01402-2, PMID: 35410119 PMC9004186

[14] Natoli JL Manack A Dean B Butler Q Turkel CC Stovner L. Global prevalence of chronic migraine: a systematic review. Cephalalgia. (2010) 30:599–609. doi: 10.1111/j.1468-2982.2009.01941.x, PMID: 19614702

[15] Hakim AJ Grahame R. Non-musculoskeletal symptoms in joint hypermobility Syndrome. Indirect evidence for autonomic dysfunction? Rheumatology (Oxford). (2004) 43:1194–5. doi: 10.1093/rheumatology/keh279 15317957

[16] Kanjwal K Saeed B Karabin B Kanjwal Y Grubb BP. Comparative clinical profile of postural orthostatic tachycardia patients with and without joint hypermobility Syndrome. Indian Pacing Electrophysiol J. (2010) 10:173–8. PMID: 20376184 PMC2847867

[17] Bendik EM Tinkle BT Al-shuik E Levin L Martin A Thaler R. Joint hypermobility Syndrome: a common clinical disorder associated with migraine in women. Cephalalgia. (2011) 31:603–13. doi: 10.1177/0333102410392606, PMID: 21278238

[18] Fairweather D Bruno KA Darakjian AA Bruce BK Gehin JM Kotha A. High overlap in patients diagnosed with Hypermobile Ehlers-Danlos Syndrome or Hypermobile Spectrum disorders with fibromyalgia and 40 self-reported symptoms and comorbidities. Front Med (Lausanne). (2023) 10:1096180. doi: 10.3389/fmed.2023.1096180, PMID: 37181352 PMC10166812

[19] Puledda F Viganò A Celletti C Petolicchio B Toscano M Vicenzini E. A study of migraine characteristics in joint hypermobility Syndrome a.K.A. Ehlers-Danlos Syndrome, hypermobility type. Neurol Sci. (2015) 36:1417–24. doi: 10.1007/s10072-015-2173-6, PMID: 25791889

[20] Cevoli S Giannini G Favoni V Terlizzi R Sancisi E Nicodemo M. Treatment of withdrawal headache in patients with medication overuse headache: a pilot study. J Headache Pain. (2017) 18:56. doi: 10.1186/s10194-017-0763-9, PMID: 28500492 PMC5429287

[21] Croop R Berman G Kudrow D Mullin K Thiry A Lovegren M. A multicenter, open-label long-term safety study of Rimegepant for the acute treatment of migraine. Cephalalgia. (2024) 44:3331024241232944. doi: 10.1177/03331024241232944, PMID: 38659334

[22] Krymchantowski A Jevoux C Krymchantowski AG Ramos LB Barbosa JSS Silva-Neto RP. Medication-overuse headache-a review of different treatment strategies. Front Pain Res (Lausanne). (2023) 4:1103497. doi: 10.3389/fpain.2023.1103497, PMID: 37881687 PMC10597723

[23] Dobrocky T Nicholson P Häni L Mordasini P Krings T Brinjikji W. Spontaneous intracranial hypotension: searching for the Csf leak. Lancet Neurol. (2022) 21:369–80. doi: 10.1016/s1474-4422(21)00423-3, PMID: 35227413

[24] Neilson D Martin VT. Joint hypermobility and headache: understanding the glue that binds the two together--part 1. Headache. (2014) 54:1393–402. doi: 10.1111/head.12418 25040892

[25] Roma M Marden CL De Wandele I Francomano CA Rowe PC. Postural tachycardia Syndrome and other forms of orthostatic intolerance in Ehlers-Danlos Syndrome. Auton Neurosci. (2018) 215:89–96. doi: 10.1016/j.autneu.2018.02.006 29519641

[26] Doolan BJ Lavallee ME Hausser I Schubart JR Michael Pope F Seneviratne SL. Extracutaneous features and complications of the Ehlers-Danlos syndromes: a systematic review. Front Med (Lausanne). (2023) 10:1053466. doi: 10.3389/fmed.2023.1053466, PMID: 36756177 PMC9899794

[27] Gazit Y Nahir AM Grahame R Jacob G. Dysautonomia in the joint hypermobility Syndrome. Am J Med. (2003) 115:33–40. doi: 10.1016/s0002-9343(03)00235-3 12867232

[28] Song B Yeh P Harrell J. Systemic manifestations of Ehlers-Danlos Syndrome. Proc (Baylor Univ Med Cent). (2020) 34:49–53. doi: 10.1080/08998280.2020.1805714, PMID: 33456144 PMC7785142

[29] Khurana RK Eisenberg L. Orthostatic and non-orthostatic headache in postural tachycardia Syndrome. Cephalalgia. (2011) 31:409–15. doi: 10.1177/0333102410382792, PMID: 20819844

[30] Sandroni P Opfer-Gehrking TL McPhee BR Low PA. Postural tachycardia Syndrome: clinical features and follow-up study. Mayo Clin Proc. (1999) 74:1106–10. doi: 10.4065/74.11.1106, PMID: 10560597

[31] Li H Yu X Liles C Khan M Vanderlinde-Wood M Galloway A. Autoimmune basis for postural tachycardia Syndrome. J Am Heart Assoc. (2014) 3:e000755. doi: 10.1161/jaha.113.000755, PMID: 24572257 PMC3959717

[32] Heyer GL Fedak EM LeGros AL. Symptoms predictive of postural tachycardia Syndrome (Pots) in the adolescent headache patient. Headache. (2013) 53:947–53. doi: 10.1111/head.12103, PMID: 23574111

[33] Ray JC Pham X Foster E Cheema S Corcoran SJ Matharu MS. The prevalence of headache disorders in postural tachycardia Syndrome: a systematic review and Meta-analysis of the literature. Cephalalgia. (2022) 42:1274–87. doi: 10.1177/03331024221095153, PMID: 35469447

[34] Hakim A O'Callaghan C De Wandele I Stiles L Pocinki A Rowe P. Cardiovascular autonomic dysfunction in Ehlers-Danlos Syndrome-Hypermobile type. Am J Med Genet C: Semin Med Genet. (2017) 175:168–74. doi: 10.1002/ajmg.c.31543, PMID: 28160388

[35] De Wandele I Rombaut L Leybaert L Van de Borne P De Backer T Malfait F. Dysautonomia and its underlying mechanisms in the hypermobility type of Ehlers-Danlos Syndrome. Semin Arthritis Rheum. (2014) 44:93–100. doi: 10.1016/j.semarthrit.2013.12.006, PMID: 24507822

[36] Miller AJ Raj SR. Pharmacotherapy for postural tachycardia Syndrome. Auton Neurosci. (2018) 215:28–36. doi: 10.1016/j.autneu.2018.04.008 29753556

[37] Schievink WI Maya MM Moser F Tourje J Torbati S. Frequency of spontaneous intracranial hypotension in the emergency department. J Headache Pain. (2007) 8:325–8. doi: 10.1007/s10194-007-0421-8, PMID: 18071632 PMC3476164

[38] Reinstein E Pariani M Bannykh S Rimoin DL Schievink WI. Connective tissue Spectrum abnormalities associated with spontaneous cerebrospinal fluid leaks: a prospective study. Eur J Hum Genet. (2013) 21:386–90. doi: 10.1038/ejhg.2012.191 22929030 PMC3598315

[39] Cheema S Anderson J Angus-Leppan H Armstrong P Butteriss D Carlton Jones L. Multidisciplinary consensus guideline for the diagnosis and Management of Spontaneous Intracranial Hypotension. J Neurol Neurosurg Psychiatry. (2023) 94:835–43. doi: 10.1136/jnnp-2023-331166, PMID: 37147116 PMC10511987

[40] Goldberg J Häni L Jesse CM Zubak I Piechowiak EI Gralla J. Spontaneous intracranial hypotension without Csf leakage-concept of a pathological cranial to spinal fluid shift. Front Neurol. (2021) 12:760081. doi: 10.3389/fneur.2021.760081, PMID: 34790164 PMC8591068

[41] Schievink WI Maya M Prasad RS Wadhwa VS Cruz RB Moser FG. Spontaneous spinal cerebrospinal fluid-venous fistulas in patients with orthostatic headaches and Normal conventional brain and spine imaging. Headache. (2021) 61:387–91. doi: 10.1111/head.14048, PMID: 33484155

[42] D'Antona L Jaime Merchan MA Vassiliou A Watkins LD Davagnanam I Toma AK. Clinical presentation, investigation findings, and treatment outcomes of spontaneous intracranial hypotension Syndrome: a systematic review and Meta-analysis. JAMA Neurol. (2021) 78:329–37. doi: 10.1001/jamaneurol.2020.4799, PMID: 33393980 PMC7783594

[43] Häni L Fung C Jesse CM Ulrich CT Piechowiak EI Gralla J. Outcome after surgical treatment of cerebrospinal fluid leaks in spontaneous intracranial hypotension-a matter of time. J Neurol. (2022) 269:1439–46. doi: 10.1007/s00415-021-10710-7, PMID: 34274993 PMC8857147

[44] Claybrooks R Kayanja M Milks R Benzel E. Atlantoaxial fusion: a biomechanical analysis of two C1-C2 fusion techniques. Spine J. (2007) 7:682–8. doi: 10.1016/j.spinee.2006.08.010, PMID: 17434809

[45] Vaccaro AR Lim MR Lee JY. Indications for surgery and stabilization techniques of the Occipito-cervical junction. Injury. (2005) 36:B44–53. doi: 10.1016/j.injury.2005.06.014, PMID: 15993117

[46] Helgeson MD Lehman RA Jr Sasso RC Dmitriev AE Mack AW Riew KD. Biomechanical analysis of Occipitocervical stability afforded by three fixation techniques. Spine J. (2011) 11:245–50. doi: 10.1016/j.spinee.2011.01.021, PMID: 21377608

[47] Mao G Kopparapu S Jin Y Davidar AD Hersh AM Weber-Levine C. Craniocervical instability in patients with Ehlers-Danlos Syndrome: controversies in diagnosis and management. Spine J. (2022) 22:1944–52. doi: 10.1016/j.spinee.2022.08.008, PMID: 36028216

[48] Henderson FC Francomano CA Koby M Tuchman K Adcock J Patel S. Cervical medullary Syndrome secondary to Craniocervical instability and ventral brainstem compression in hereditary hypermobility connective tissue disorders: 5-year follow-up after Craniocervical reduction, fusion, and stabilization. Neurosurg Rev. (2019) 42:915–36. doi: 10.1007/s10143-018-01070-4, PMID: 30627832 PMC6821667

[49] Henderson FC Sr Henderson FC Jr WAT W Mark AS Koby M. Utility of the Clivo-axial angle in assessing brainstem deformity: pilot study and literature review. Neurosurg Rev. (2018) 41:149–63. doi: 10.1007/s10143-017-0830-3, PMID: 28258417 PMC5748419

[50] Lohkamp L-N Marathe N Fehlings MG. Craniocervical instability in Ehlers-Danlos Syndrome—a systematic review of diagnostic and surgical treatment criteria. Global Spine J. (2022) 12:1862–71. doi: 10.1177/21925682211068520, PMID: 35195459 PMC9609512

[51] Russek LN Block NP Byrne E Chalela S Chan C Comerford M. Presentation and physical therapy Management of Upper Cervical Instability in patients with symptomatic generalized joint hypermobility: international expert consensus recommendations. Front Med (Lausanne). (2022) 9:1072764. doi: 10.3389/fmed.2022.1072764, PMID: 36743665 PMC9893781

[52] Society IH. 11.2.1 Cervicogenic headache: International (2021). 3. Available from: https://ichd-3.org/11-headache-or-facial-pain-attributed-to-disorder-of-the-cranium-neck-eyes-ears-nose-sinuses-teeth-mouth-or-other-facial-or-cervical-structure/11-2-headache-attributed-to-disorder-of-the-neck/11-2-1-cervicogenic-headache/ (Accessed on 12th of May 2022)

[53] Martin VT Neilson D. Joint hypermobility and headache: the glue that binds the two together--part 2. Headache. (2014) 54:1403–11. doi: 10.1111/head.12417 24958300

[54] Al Khalili Y Ly N Murphy PB. Cervicogenic headache. Statpearls. Treasure Island (FL): Stat pearls publishing copyright © 2024, stat pearls publishing LLC (2024). 29939639

[55] Calhoun AH Ford S Millen C Finkel AG Truong Y Nie Y. The prevalence of neck pain in migraine. Headache. (2010) 50:1273–7. doi: 10.1111/j.1526-4610.2009.01608.x 20100298

[56] Dwyer A Aprill C Bogduk N. Cervical zygapophyseal joint pain patterns. I: A Study in Normal Volunteers Spine (Phila Pa 1976). (1990) 15:453–7. doi: 10.1097/00007632-199006000-00004 2402682

[57] Bogduk N Govind J. Cervicogenic headache: an assessment of the evidence on clinical diagnosis, invasive tests, and treatment. Lancet Neurol. (2009) 8:959–68. doi: 10.1016/s1474-4422(09)70209-1, PMID: 19747657

[58] Coskun O Ucler S Karakurum B Atasoy HT Yildirim T Ozkan S. Magnetic resonance imaging of patients with Cervicogenic headache. Cephalalgia. (2003) 23:842–5. doi: 10.1046/j.1468-2982.2003.00605.x 14510932

[59] Maini K Dua A. Temporomandibular Syndrome. Statpearls. Treasure Island (FL): Stat pearls publishing copyright © 2024, stat pearls publishing LLC (2024). 31869076

[60] Lomas J Gurgenci T Jackson C Campbell D. Temporomandibular dysfunction. Aust J Gen Pract. (2018) 47:212–5. doi: 10.31128/afp-10-17-4375 29621862

[61] Saruhanoğlu A Gökçen-Röhlig B Saruhanoğlu C Öngül D Koray M. Frequency of temporomandibular disorder signs and symptoms among call center employees. Cranio. (2017) 35:244–9. doi: 10.1080/08869634.2016.1216823, PMID: 27684502

[62] Gonçalves DA Camparis CM Speciali JG Franco AL Castanharo SM Bigal ME. Temporomandibular disorders are differentially associated with headache diagnoses: a controlled study. Clin J Pain. (2011) 27:611–5. doi: 10.1097/AJP.0b013e31820e12f5, PMID: 21368664

[63] Talmaceanu D Lenghel LM Bolog N Hedesiu M Buduru S Rotar H. Imaging modalities for temporomandibular joint disorders: an update. Clujul Med. (2018) 91:280–7. doi: 10.15386/cjmed-970, PMID: 30093805 PMC6082607

[64] Gauer RL Semidey MJ. Diagnosis and treatment of temporomandibular disorders. Am Fam Physician. (2015) 91:378–86. 25822556

[65] Strahle J Muraszko KM Kapurch J Bapuraj JR Garton HJ Maher CO. Chiari malformation type I and Syrinx in children undergoing magnetic resonance imaging. J Neurosurg Pediatr. (2011) 8:205–13. doi: 10.3171/2011.5.Peds1121 21806364

[66] Speer MC Enterline DS Mehltretter L Hammock P Joseph J Dickerson M. Review article: Chiari type I malformation with or without Syringomyelia: prevalence and genetics. J Genet Couns. (2003) 12:297–311. doi: 10.1023/a:1023948921381 26141174

[67] Schijman E. History, anatomic forms, and pathogenesis of Chiari I malformations. Childs Nerv Syst. (2004) 20:323–8. doi: 10.1007/s00381-003-0878-y, PMID: 14762679

[68] Milhorat TH Bolognese PA Nishikawa M McDonnell NB Francomano CA. Syndrome of Occipitoatlantoaxial hypermobility, cranial settling, and Chiari malformation type I in patients with hereditary disorders of connective tissue. J Neurosurg Spine. (2007) 7:601–9. doi: 10.3171/spi-07/12/601, PMID: 18074684

[69] Clarke JE Reyes JM Luther E Govindarajan V Leuchter JD Niazi T. Chiari I malformation Management in Patients with heritable connective tissue disorders. World Neurosurg X. (2023) 18:100173. doi: 10.1016/j.wnsx.2023.100173, PMID: 36969375 PMC10031113

[70] Milhorat TH Chou MW Trinidad EM Kula RW Mandell M Wolpert C. Chiari I malformation redefined: clinical and radiographic findings for 364 symptomatic patients. Neurosurgery. (1999) 44:1005–17. doi: 10.1097/00006123-199905000-00042, PMID: 10232534

[71] Bhadelia RA Frederick E Patz S Dubey P Erbay SH Do-Dai D. Cough-associated headache in patients with Chiari I malformation: Csf flow analysis by means of cine phase-contrast Mr imaging. AJNR Am J Neuroradiol. (2011) 32:739–42. doi: 10.3174/ajnr.A2369, PMID: 21330393 PMC7965870

[72] Urbizu A Toma C Poca MA Sahuquillo J Cuenca-León E Cormand B. Chiari malformation type I: a case-control association study of 58 developmental genes. PLoS One. (2013) 8:e57241. doi: 10.1371/journal.pone.0057241, PMID: 23437350 PMC3578784

[73] Massimi L Peretta P Erbetta A Solari A Farinotti M Ciaramitaro P. Diagnosis and treatment of Chiari malformation type 1 in children: the international consensus document. Neurol Sci. (2022) 43:1311–26. doi: 10.1007/s10072-021-05317-9, PMID: 34097175 PMC8789635

[74] Panigrahi M Reddy BP Reddy AK Reddy JJ. Csf flow study in Chiari I malformation. Childs Nerv Syst. (2004) 20:336–40. doi: 10.1007/s00381-003-0881-3 15085382

[75] McGirt MJ Nimjee SM Fuchs HE George TM. Relationship of cine phase-contrast magnetic resonance imaging with outcome after decompression for Chiari I malformations. Neurosurgery. (2006) 59:140–6. doi: 10.1227/01.neu.0000243293.46319.35, PMID: 16823310

[76] Kim ST Brinjikji W Lanzino G Kallmes DF. Neurovascular manifestations of connective-tissue diseases: a review. Interv Neuroradiol. (2016) 22:624–37. doi: 10.1177/1591019916659262 27511817 PMC5564353

[77] Grande RB Aaseth K Lundqvist C Russell MB. Prevalence of new daily persistent headache in the general population. The Akershus study of chronic headache. Cephalalgia. (2009) 29:1149–55. doi: 10.1111/j.1468-2982.2009.01842.x 19830882

[78] Rozen TD Roth JM Denenberg N. Cervical spine joint hypermobility: a possible predisposing factor for new daily persistent headache. Cephalalgia. (2006) 26:1182–5. doi: 10.1111/j.1468-2982.2006.01187.x, PMID: 16961783

[79] Cheema S Stubberud A Rantell K Nachev P Tronvik E Matharu M. Phenotype of new daily persistent headache: subtypes and comparison to transformed chronic daily headache. J Headache Pain. (2023) 24:109. doi: 10.1186/s10194-023-01639-5, PMID: 37587430 PMC10428606

[80] Yamani N Olesen J. 12th European headache federation congress jointly with 32nd National Congress of the Italian Society for the Study of headaches. Ew Daily Persistent Headache: Systematic Rev Enigmatic Disor. (2018) 19:1–147. doi: 10.1186/s10194-018-0900-0

[81] Cheema S Mehta D Ray JC Hutton EJ Matharu MS. New daily persistent headache: a systematic review and Meta-analysis. Cephalalgia. (2023) 43:033310242311680. doi: 10.1177/03331024231168089 37032616

